# Trends in Incisional and Ventral Hernia Repair: A Population Analysis From 2001 to 2021

**DOI:** 10.7759/cureus.35744

**Published:** 2023-03-03

**Authors:** Madeline Gillies, Lakmali Anthony, Aymen Al-Roubaie, Aaron Rockliff, Jenny Phong

**Affiliations:** 1 General Surgery, Goulburn Valley Health, Shepparton, AUS; 2 Vascular Surgery, Northern Hospital Epping, Epping, AUS; 3 General Surgery, Western Health, Melbourne, AUS; 4 General Surgery, Northern Hospital Epping, Epping, AUS

**Keywords:** painful and irreducible or incarcerated hernias, day surgery, elective ivhr, australian bureau of statistics, australian institute of health and welfare, general emergency surgery, emergency hernia repair, strangulated hernia, hernia mesh, ventral and incisional hernia

## Abstract

Background

Incisional and ventral hernias are highly prevalent, with primary ventral hernias occurring in approximately 20% of adults and incisional hernias developing in up to 30% of midline abdominal incisions. Recent data from the United States have shown an increasing incidence of elective incisional and ventral hernia repair (IVHR) and emergency repair of complicated hernias. This study examines Australian population trends in IVHR over a two-decade study period.

Methods

This retrospective study was performed using procedure data from the Australian Institute of Health and Welfare and population data from the Australian Bureau of Statistics captured between 2000 and 2021 to calculate incidence rates per 100,000 population by age and sex for selected subcategories of IVHR operations. Trends over time were evaluated using simple linear regression.

Results

There were 809,308 IVHR operations performed in Australia during the study period. The cumulative incidence adjusted for population was 182 per 100,000; this increased by 9.578 per year during the study period (95%CI = 8.431-10.726, p<.001). IVHR for primary umbilical hernias experienced the most significant increase in population-adjusted incidence, 1.177 per year (95%CI = 0.654-1.701, p<.001). Emergency IVHR for incarcerated, obstructed, and strangulated hernias increased by 0.576 per year (95%CI = 0.510-0.642, p<.001). Only 20.2% of IVHR procedures were performed as day surgery.

Conclusions

Australia has seen a significant increase in IVHR operations performed in the last 20 years, particularly those for primary ventral hernias. IVHR for hernias complicated by incarceration, obstruction, and strangulation also increased significantly. The proportion of IVHR operations performed as day surgery is well below the target set by the Royal Australasian College of Surgeons. With the increasing incidence of IVHR operations and an increasing proportion of these being emergent, elective IVHR should be performed as day surgery when it is safe.

## Introduction

Incisional and ventral hernias are common conditions, and repair of these hernias is ubiquitous in the practice of general surgery. Primary ventral hernias occur in approximately one in five adults, and incisional hernias develop in 10-30% of midline abdominal incisions [1,2]. Furthermore, up to 14.4% of primary ventral hernias and 32% of incisional hernias result in recurrence [3]. The risk of incisional and ventral hernia development, as well as the risk of incarceration, obstruction, strangulation, and recurrence increases with BMI and age [3,4]. Recurrent and complicated hernias significantly increase morbidity, mortality, and length of hospital stay [3]. The ageing population, together with the increasing prevalence of obesity in Australia, means that incisional and ventral hernia repair (IVHR) operations are likely to increase significantly over time [5,6]. Previous studies in the United States have indicated that the incidence of IVHR operations is rising, and the proportion of these procedures undertaken emergently is increasing [7]. However, no studies have characterised the incidence of IVHR in an Australian population. As such, this study aims to describe the incidence of IVHR operations, as well as the proportion of these performed emergently and as day surgery.

## Materials and methods

Data source

This study used data from the Australian Institute of Health and Welfare (AIHW) Procedures Data Cubes from the National Hospitals Data Collection. Data were extracted from procedure chapter X 'Procedures on Digestive System', subcategory: ‘Operations on Abdomen/Peritoneum/Omentum’, and blocks 0992 ‘Repair of Umbilical, Epigastric or Linea Alba Hernia’, ‘0993 - Repair of Incisional Hernia’, ‘0994-Repair of Parastomal Hernia’, and ‘0996 - Repair of Other Abdominal Wall Hernias’. These data do not separate laparoscopic from open IVHR. The population-adjusted incidence of IVHR (per 100,000 population) was calculated by dividing the unadjusted annual incidence by the population estimate of the Australian Bureau of Statistics (ABS) for that year (by age group and sex, where appropriate). This study did not require ethics board approval, since publicly available data was used.

Statistical analysis

The unadjusted incidence of total IVHR and selected subcategories are presented with descriptive statistics. The population-adjusted incidence was calculated per 100,000 population using annual ABS data for each age and sex group. Simple linear regression was performed to quantify the change in population-adjusted incidence of IVHR with year as the independent variable. This was expressed as a change per year per 100,000 people (R^2^) with 95% confidence.

A p-value less than 0.05 was considered statistically significant. All statistical analyses were performed with IBM SPSS Statistics for Windows, Version 28.0 (Released 2021; IBM Corp., Armonk, New York, United States), and figures were generated using GraphPad Prism 8 (Dotmatics, Boston, Massachusetts, United States).

## Results

During 2000-2021, a total of 809,308 IVHR operations were performed in Australia (Table 1). Of these, 319,556 (39.4%) were umbilical, 35,353 (4.3%) were epigastric, 151,509 (18.7%) were other ventral hernias, 175,369 (21.6%) were incisional, and 17,417 (2.1%) were parastomal. Most hernias were uncomplicated at the time of repair; however, 110,105 (13.6%) were complicated by incarceration or bowel obstruction and 3,550 (1.08%) were complicated by strangulation that required concurrent bowel resection. Regarding repair method, 228,710 (96.3%) IVHR operations used prosthetic mesh, and 5,265 (2.2%) used a myocutaneous flap. The use of mesh prosthesis was approximately equal between incisional (121,754, 69.4%) and ventral (106,956, 70.6%) hernias. Myocutaenous flap was more commonly used in incisional (3,405, 2.7%) rather than ventral hernias (1,860, 1.7%). Most IVHR operations required overnight admission to hospital (640,672, 79.2%). Most of the 168,638 IVHR operations performed as a day surgery were epigastric (15,504, 43.9%) or umbilical (108,641, 34.0%), and very few (624, 3.6%) were parastomal hernias. Males comprised 58.7% of all IVHR operations during the study period and were also over-represented in umbilical (208,247, 65.2%), epigastric (20,383, 57.7%), other ventral 84,276 (55.6%), parastomal (9,252, 53.1%), and incarcerated or obstructed hernias (75,590, 68.7%). However, females comprised a slight majority of incisional hernias (97,765, 55.7%) and strangulated hernias that required bowel resection (2,188, 61.6%).

**Table 1:** Cumulative incidence of incisional and ventral hernia repair operations performed over the study period by repair method, admission type, age, and sex. * Some categories not available due to coding of AIHW data. AIHW: Australian Institute of Health and Welfare

	Umbilical	Epigastric	Other ventral	Incisional	Parastomal	Incarcerated or obstructed	Strangulated	Total
Total	319,556 (39.4%)	35,353 (4.3%)	151,509 (18.7%)	175,369 (21.6%)	17,417 (2.1%)	110,105 (13.6%)	3550 (1.08%)	809,308 (100%)
Repair								
Mesh			106,956 (70.6%)	121,754 (69.4%)				228,710 (96.3%)
Flap			1,860 (1.7%)	3,405 (2.7%)				5,265 (2.2%)
Admission type								
Day	108,641 (34.0%)	15,504 (43.9%)	21,394 (14.1%)	10,191 (5.8%)	624 (3.6%)	12,284 (11.2%)	290 (8.2%)	168,638 (20.8%)
Overnight	210,915 (66%)	19,849 (56.1%)	13,0116 (85.9%)	16,5178 (94.2%)	16,793 (96.4%)	97,821 (88.8%)	3,260 (91.8%)	640,672 (79.2%)
Age group								
<4 years	16,835 (5.3%)	2,396 (6.8%)	1,432 (0.9%)	389 (0.2%)	72 (0.4%)	3,018 (2.7%)	15 (0.4%)	24,142 (3.0%)
5 - 14 years	9,720 (3.0%)	2,253 (6.4%)	881 (0.6%)	187 (0.1%)	23 (0.1%)	1,073 (1.0%)	8 (0.2%)	14,137 (1.7%)
15 - 34 years	33,185 (10.4%)	5,188 (14.7%)	11,810 (7.8%)	7,073 (4.0%)	465 (2.7%)	9,126 (8.3%)	121 (3.4%)	66,847 (8.3%)
35 - 54 years	125,165 (39.2%)	13,317 (37.7%)	53,507 (35.3%)	48,666 (27.8%)	3,040 (17.5%)	29,478 (26.8%)	785 (22.1%)	273,173 (33.8%)
55 - 64 years	58,557 (18.3%)	6,238 (17.6%)	35,096 (23.2%)	41,819 (23.8%)	4,029 (23.1%)	20,264 (18.4%)	764 (21.5%)	166,003 (20.5%)
65 - 74 years	50,564 (15.8%)	4,031 (11.4%)	30,253 (20.0%)	46,415 (26.5%)	5,332 (30.6%)	20,526 (18.6%)	870 (24.5%)	157,121 (19.4%)
>75 years	25,530 (8%)	1,930 (5.5%)	18,530 (12.2%)	30,820 (17.6%)	4,456 (25.6%)	26,619 (24.2%)	987 (27.8%)	107,885 (13.3%)
Sex								
Male	208,247 (65.2%)	20,383 (57.7%)	84,276 (55.6%)	77,604 (44.3%)	9,252 (53.1%)	75,590 (68.7%)	1,362 (38.4%)	475,352 (58.7%)
Female	111,309 (34.8%)	14,970 (42.3%)	67,233 (44.4%)	97,765 (55.7%)	8,165 (46.9%)	34,515 (31.3%)	2,188 (61.6%)	333,957 (41.3%)

Cumulative population-adjusted incidence of IVHR

**Figure 1:**
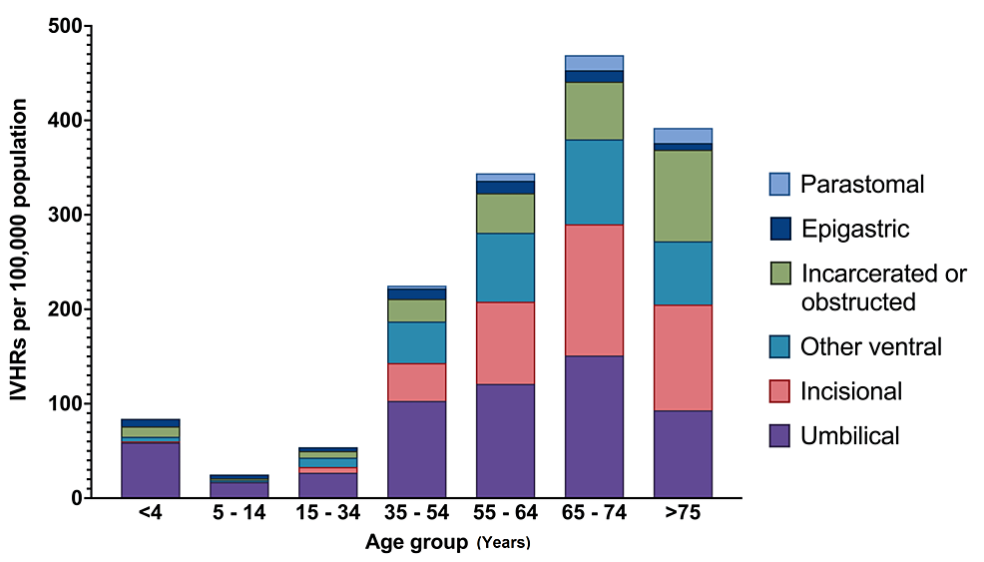
Population-adjusted cumulative incidence of incisional and ventral hernia repair for each age group by type of hernia. IVHRs: incisional and ventral hernia repairs

The estimated total Australian population ranged from 19 million in 2000 to 26.9 million in 2020. The cumulative population-adjusted incidence of IVHR was 182 per 100,000 (Table 2). The incidence varied by hernia type and was highest for umbilical hernias (72 per 100,000), followed by incisional hernias (39 per 100,000), other ventral hernias (34 per 100,000), epigastric hernias (eight per 100,000), and finally parastomal hernias (four per 100,000). The cumulative population-adjusted incidence of obstructed or incarcerated hernia was 25 per 100,000 and 0.8 per 100,000 for strangulated hernias. Males had a higher incidence of IVHR than females (221 and 153 per 100,000, respectively). The cumulative population-adjusted incidence also varied by age and was lowest in the age group of 5-14 years (25 per 100,000) and highest in the age group of 65-74 years (470 per 100,000). The cumulative population-adjusted incidence of IVHR for each age group by type of hernia is shown in Figure 1.

**Table 2:** Cumulative incidence of incisional and ventral hernia repair operations adjusted per 100,000 population for each hernia type, by age and sex.

	Umbilical	Epigastric	Other ventral	Incisional	Parastomal	Incarcerated or obstructed	Strangulated	Total
Total	72	8	34	39	4	25	0.8	182
Age group								
<4 years	59	8	5	1	0.0	11	0.1	84
5 - 14 years	17	4	2	0.0	0.0	2	0.0	25
15 - 34 years	27	4	10	6	0.0	7	0.1	54
35 - 54 years	103	11	44	40	3	24	0.6	225
55 - 64 years	121	13	73	87	8	42	1.6	344
65 - 74 years	151	12	90	139	16	61	2.6	470
>75 years	93	7	67	112	16	97	3.6	392
Sex								
Male	97	9	39	36	4	35	0.6	221
Female	51	7	31	45	4	16	1	153

Population-adjusted incidence of IVHR over time

The population-adjusted incidence of the IVHR procedure ranged from a minimum of 139 per 100,000 in the 2001-2002 financial year to a peak of 219 per 100,000 in the 2015-2016 financial year. Simple linear regression analysis showed that the population-adjusted incidence of IVHR increased by 9.578 per year during the study period (95%CI = 8.431, 10.726, p <0.001) (Table 3).

**Table 3:** Simple linear regression of annual population-adjusted incidence of incisional and ventral hernia repair operations with year as the independent variable. *Emergency operations for incarcerated, obstructed or strangulated hernias. SE: standard error

	R^2^	β	SE	95% Confidence Interval	p-value
Hernia type					
All	0.945	9.578	0.546	8.431, 10.726	<0.001
Umbilical	0.554	1.177	0.249	0.654, 1.701	<0.001
Epigastric	0.015	-0.022	0.041	-0.108, 0.065	0.602
Other ventral	0.970	1.779	0.074	1.624, 1.934	<0.001
Incisional	0.384	0.271	0.081	0.101, 0.442	0.004
Parastomal	0.888	0.281	0.024	0.232, 0.331	<0.001
Emergency*	0.949	0.576	0.032	0.510, 0.642	<0.001
Repair					
Mesh	0.888	1.792	0.150	1.477, 2.106	<0.001
Flap	0.888	1.787	0.150	1.473, 2.101	<0.001
Admission type					
Day case	0.927	0.991	0.066	0.853, 1.129	<0.001
Overnight	0.836	3.062	0.320	2.389, 3.734	<0.001
Sex					
Male	0.826	5.890	0.636	4.553, 7.227	<0.001
Female	0.947	3.535	0.196	3.123, 3.948	<0.001
Age group					
<4 years	0.957	-3.017	0.151	-3.335, -2.699	<0.001
5 - 14 years	0.001	-0.007	0.070	-0.155, 0.140	0.92
15 - 34 years	0.795	1.209	0.145	0.905, 1.512	<0.001
35 - 54 years	0.922	5.673	0.388	4.857, 6.489	<0.001
55 - 64 years	0.299	4.745	1.714	1.144, 8.346	0.013
65 - 74 years	0.815	9.573	1.075	7.315, 11.831	<0.001
>75 years	0.898	6.649	0.529	5.538, 7.760	<0.001

**Figure 2:**
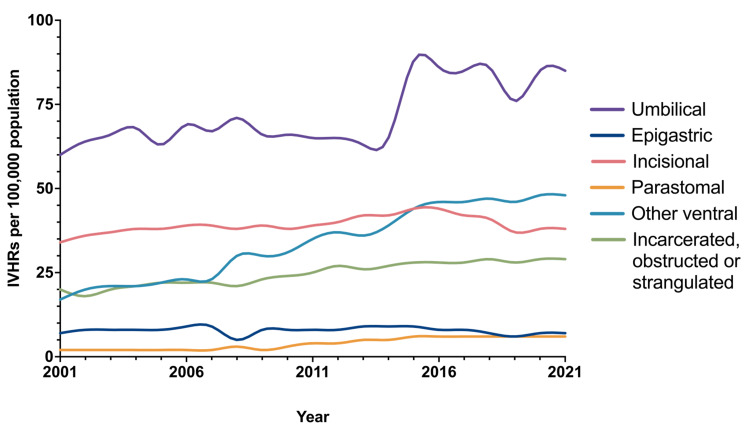
Population-adjusted incidence of incisional and ventral hernia repair over time for each type of hernia. IVHRs: incisional and ventral hernia repairs

A statistically significant increase in the annual population-adjusted incidence was found for each type of hernia, except epigastric (Table 3 and Figure 2).

**Figure 3:**
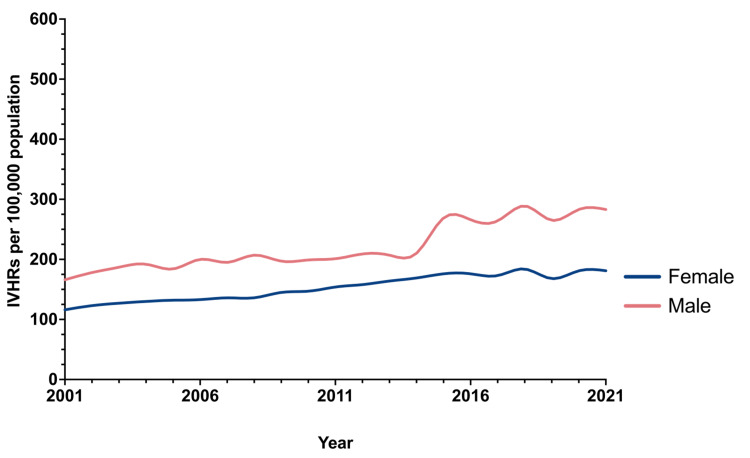
Population-adjusted incidence of incisional and ventral hernia repair over time by sex. IVHRs: incisional and ventral hernia repairs

Primary hernias had the highest growth per year, where umbilical hernias increased by 1.177 per 100,000 per year (95%CI = 0.654, 1.701, p <0.001) and other ventral hernias increased by 1.779 per 100,000 population per year (95%CI = 1.624, 1.934, p <0.001) (Table 3 and Figure 2). Incisional hernias had a relatively low increase (0.271, 95%CI = 0.101, 0.442, p=0.004), as did parastomal hernias (0.281, 95%CI = 0.232, 0.331, p <0.001) (Table 3 and Figure 2). Population-adjusted incidence of IVHR operations for incarcerated, obstructed, and strangulated hernias increased by 0.576 per year (95%CI = 0.510, 0.642, p <0.001) (Table 3). Regarding the repair method, the population-adjusted incidence of IVHR operations using prosthetic mesh and those using myocutaneous flap increased at a similar rate, where mesh repairs increased by 1.792 per year (95%CI = 1.477, 2.106, p <0.001) and those using myocutaneous flap increased by 1.787 per year (95%CI = 1.473, 2.101, p <0.001) (Table 3). Both male and female patients experienced an increased incidence of IVHR during the study period; however, this increase was greater for males (Figure 3).

**Figure 4:**
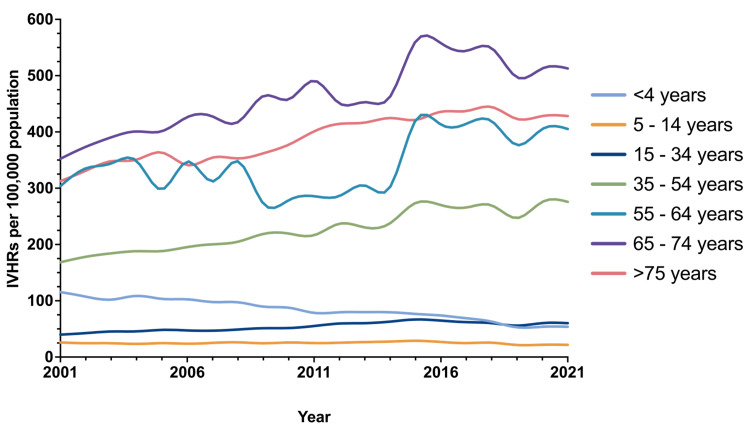
Population-adjusted incidence of incisional and ventral hernia repair over time by age group. IVHRs: incisional and ventral hernia repairs

The population-adjusted incidence of IVHR operations in male patients increased by 5.890 per year (95%CI = 4.553, 7.227, p <0.001), while female patients saw an annual increase of 3.535 (95%CI = 3.113, 3.948, p <0.001)(Table 3). The population-adjusted incidence of IVHR over the study period by age group is shown in Figure 4.

Most age groups experienced an increased incidence of IVHR, except for the paediatric group under four years (Table 3). In this group, the annual population-adjusted incidence of IVHR decreased by 3.017 (95%CI = -3.335, -2.699, p <0.001) (Table 3). A decrease of 0.007 per 100,000 per year was observed in the other paediatric group (5-14 years); however, this was not statistically significant (95%CI = -0.155, 0.140, p=0.92). Patients in the age group of 65-74 years had the greatest increase in population-adjusted incidence of IVHR, 9.573 per year (95%CI = 7.315, 11.831, p <0.001).

## Discussion

This study has shown that IVHR operations in Australia have increased during the 20-year study period. Similar findings have been reported in recent data from the United States [8]. A possible explanation is the increasing incidence of obesity in Australia [5]. Obesity is a risk factor for the development and recurrence of primary and incisional ventral hernias due to increased intraabdominal pressure, increased visceral fat, and increased risk of surgical site infection [4]. In contrast to IVHR, recent data suggest that the number of inguinal hernia operations performed in Australia is decreasing [9]. This may be because, unlike incisional and ventral hernias, the risk of inguinal hernia may not increase due to obesity, and high BMI may even have a protective effect on the risk of inguinal hernia [10]. A second explanation for the increase in incisional and ventral hernias and the decrease in inguinal hernia repair is a change in clinical guidelines, which discourages the repair of asymptomatic inguinal hernias [11]. Royal Australasian College of Surgeons (RACS) recommends that surgical repair of minimally or asymptomatic inguinal hernias must be carefully considered [11]. A randomised controlled trial investigating the watchful waiting approach in incisional and ventral hernias is ongoing [12]. As such, consensus guidelines for the treatment of asymptomatic incisional and ventral hernias are not available and there remains significant variability between surgeons in operative management [13].

Hernia type

The guidelines of the European Hernia Society classify anterior abdominal wall hernias as primary or incisional and exclude inguinal and femoral groin hernias [14]. Primary hernias (or ‘ventral’) hernias can then be subclassified as midline umbilical and epigastric hernias, as well as lateral spigelian hernias [14]. Although parastomal hernias are incisional by definition, they are a distinct category of ventral hernia with different treatment options [14]. Before this consensus definition, the literature on abdominal wall hernias had been hampered by inconsistent nomenclature, and incisional and primary ventral hernias continue to be combined in studies despite distinct aetiology, epidemiology, and surgical outcomes [14]. In this study, primary ventral, incisional, and parastomal hernias were reported separately. An estimated 75% of hernias of the anterior abdominal wall are primary ventral (non-incisional), consistent with the findings of this study [15]. The slight decrease in incisional hernias towards the end of the study period coincides with the publication of the results of the STITCH trial, a landmark hernia surgery trial showing the reduced rate of incisional hernia after closure of the midline laparotomy using a small bite technique [16]. Although no data on the adoption of this technique by Australian surgeons are published and the association is unlikely to be causal, it is plausible that the dissemination of the STITCH results and other more recent research has led to a general increase in the cognisance of incisional hernia prevention techniques for midline laparotomy [1,17-19]. The increased use of minimally invasive techniques in place of large laparotomy incisions may also explain some of the variations in incisional hernias. Research that analyses the incidence of incisional hernia in the Australian population beyond 2021 and qualitative research that analyses the laparotomy closure techniques used by members of RACS over time would provide evidence for these hypotheses.

Emergency

Painful and irreducible (also known as incarcerated) hernias imply a risk of bowel obstruction and strangulation. This presentation requires an emergency operation to reduce and repair the hernia and resect the non-viable bowel in the case of strangulation. This is associated with significantly more complications, morbidity, and mortality compared to elective IVHR [20]. Previous population samples in the United States published by Beadles et al. and Colavita et al. found that emergency IVHR is required in 14.5-18.9% of cases, consistent with 13.5% of incarcerated or obstructed and 1.08% of strangulated hernias in the present study [8,21]. Both of these epidemiological studies also found that the population-adjusted incidence of emergency IVHR for complicated hernias increased over time [8,21]. A recent prospective study of 4,472 patients found that the risk of incarceration hernias increased with high BMI and older age for both primary ventral and incisional hernias [3]. The risk of incarceration was also increased by constipation in primary ventral hernias and female sex in incisional hernias [3]. The increasing incidence of emergency IVHR may be due in part to the ageing population in Australia, the increasing prevalence of obesity, and diets increasingly rich in constipating foods [5,6,22].

Repair type

This study found that the cumulative incidence of mesh use was 95.3%. The use of prosthetic mesh to reduce tension and increase repair strength is the current standard of care for IVHR. The use of mesh has been shown to reduce hernia recurrence rates in elective repair of both primary ventral and incisional hernia [19,23]. Howard et al. found that mesh use in primary ventral hernia repairs performed in the United States between 2017 and 2018 increased from 63.2% to 72.5%, which is consistent with the increased use of mesh shown in the present study [24]. A second use of mesh is in the repair of complex abdominal wall hernias. Significant fascial defects that cannot be closed primarily can be bridged with mesh (with or without component separation). In the case of an accompanying skin defect, a myocutaneous flap (most commonly rectus abdominis or tensor fascia lata) can be rotated into the defect [25]. The use of a myocutaneous flap was used in only 2.2% of IVHR operations in the present study, but increased during the study period. Howard et al. also found that the use of myocutaneous flaps increased with time and is currently used in 16.3% of IVHR in the United States [24]. However, possible differences in administrative data coding mean that these proportions may not be directly comparable.

Hospital admission

Most IVHR procedures performed during the study period required an overnight stay in hospital. Umbilical and epigastric hernias were more likely to be performed as a day surgery; however, most patients were still admitted overnight. This remained relatively stable over the study period. The 34% of umbilical hernia repairs performed as day surgery in this study falls well short of the 70-80% target suggested by the RACS guidelines. Mills et al. found a similar situation for inguinal hernia repairs performed in Australia between 2000 and 2019, which were also significantly below the RACS target, with only 22.2% performed as day surgery [26,27]. A survey of Australian patients who underwent inguinal hernia repair as a day procedure found that 88% were satisfied with their experience and would choose day surgery again [28]. The benefits of day surgery include a decrease in the risk of hospital-acquired infections, a reduced healthcare cost, and a decreased risk of cancellation due to the lack of hospital beds [26]. This finding represents a large discrepancy between guidelines and clinical practice that is relevant in the context of the current increasing waiting times for elective surgery, the decreasing ratio of hospital beds per person, and the increased access block seen in the Australian hospital system [29].

Age and sex

The incidence of IVHR between males and females was consistent with previous findings, with a cumulative incidence of most hernia types higher in males than females [8]. This was with the exception of incisional hernias, which were more common in females, which has also been previously shown [8,20]. The population-adjusted incidence of IVHR generally increased with age, with the exception of the under-four years age group, which had a higher incidence of umbilical, epigastric, and incisional hernias than the age group of 5-14 years, as well as the 15-34 years age group for umbilical and epigastric hernias. This is undoubtedly due to congenital abdominal wall defects specific to this age group. The population-adjusted incidence was highest in the age group of 65-74 years for each type of IVHR operation for simple hernias and overall. In contrast, the population-adjusted incidence of IVHR for incarcerated, obstructed, and strangulated hernias was highest in the over-75 years age group, consistent with previous findings [24].

Strengths and limitations

The main strength of this study is the large population size and the long study period facilitated by the retrospective analysis of AIHW coding data. This allowed a nationwide population analysis of IVHR, including some patient demographic data such as age and sex. As such, this article is the largest and most comprehensive overview of IVHR operations performed in Australia over the past two decades and describes previously uncharacterised population-adjusted incidence of IVHR operations in Australia. Conversely, the use of these data may also be a limitation of this article, as administrative data is less reliable than clinical data primarily because these data are collected for the purpose of management, planning, and activity monitoring of health services by administrative staff rather than for the purpose of epidemiologic research by clinical staff [30]. A further limitation of this study is the absence of some important clinical variables, such as operative technique. Although AIHW International Classification of Diseases, Tenth Revision, Clinical Modification (ICD-10-CM) codes allow for the capture of laparoscopic repair of groin hernias separately from open repair, this distinction is not available for IVHR. Similarly, the distinction between initial repair and repair of recurrent hernias would allow a description of the burden of recurrent incisional and ventral hernias in Australia.

## Conclusions

The population-adjusted incidence of IVHR operations performed in Australia has increased significantly during the 20-year study period. This was particularly so for primary ventral hernias. The incidence of incisional hernias had recently shown a downward trend; however, this was not statistically significant and would need to be re-evaluated as more data become available. Furthermore, the incidence of IVHR performed for incarceration, obstruction, and strangulation increased significantly during the study period. This is the first article to describe the concerning increase in emergency IVHR operations in the Australian population, highlighting the importance of access to surgical care for patients with incisional and ventral hernia. Finally, this study has shown that the proportion of IVHR operations performed as day surgery in Australia is well below the RACS target, highlighting the need for Australian surgeons to perform these procedures as day surgery in patients in whom it is safe to do so.
